# Supplementary material for: A Fenchone Derivative Effectively Abrogates Joint Damage Following Post-Traumatic Osteoarthritis in Lewis Rats
Source: Cells. 2022 Dec 16;11(24):4084. doi: 10.3390/cells11244084 (PMC9777073; doi:10.3390/cells11244084)
Supplement: Supplementary file 1 [file cells-11-04084-s001.zip › cells-2035988-Supplemtary data 1-6_PROOF-done.pdf]

cells

MDPI

# Supplementary Data

**SD1.** Agonist Secondary Screen.

| Compound Name | Assay Name | Assay Format | Assay Target | Result Type | RC50 | Unit | Hill | Curve Bottom | Curve Top | Max Response |
|---|---|---|---|---|---|---|---|---|---|---|
| CP55940 | cAMP | Agonist | CNR1 | EC50 | 0.0005264 | uM | 1.4688 | 6.2079 | 101.09 | 99.714 |
| CP55940 | cAMP | Agonist | CNR2 | EC50 | 0.001043 | uM | 1.3235 | 0.39811 | 98.758 | 98.466 |
| 1b | cAMP | Agonist | CNR1 | EC50 | >10 | uM | | | | 9.47 |
| 1b | cAMP | Agonist | CNR2 | EC50 | 0.05135 | uM | 0.76571 | 9.9557 | 59.136 | 58.829 |
| 1d | cAMP | Agonist | CNR1 | EC50 | >10 | uM | | | | 8.725 |
| 1d | cAMP | Agonist | CNR2 | EC50 | 0.01215 | uM | 1.0229 | 0.15957 | 92.918 | 93.13 |

**SD1. Control dose response curves for the selected GPCR Biosensor Assays:** Control dose curves were performed for the requested GPCR Biosensor Assays. Data shown was normalized to the maximal and minimal response observed in the presence of control compound and vehicle respectively. For Gi cAMP assays, the following forskolin concentration was used:CNR1: 20µM Forskolin; CNR2: 25µM Forskolin

**SD2.** Antagonist Secondary Screen.

| Compound Name | Assay Name | Assay Format | Assay Target | Result Type | RC50 | Unit | Hill | Curve Bottom | Curve Top | Max Response |
|---|---|---|---|---|---|---|---|---|---|---|
| **CP55940** | cAMP | Agonist | CNR1 | EC50 | 0.0003274 | uM | 1.0753 | -1.8765 | 100 | 101.12 |
| **AM 281** | cAMP | Antagonist | CNR1 | IC50 | 0.02237 | uM | 1.083 | 1.8776 | 100 | 100.14 |
| **CP55940** | cAMP | Agonist | CNR2 | EC50 | 0.0005945 | uM | 0.81469 | -10.872 | 100 | 100.3 |
| **SR 144528** | cAMP | Antagonist | CNR2 | IC50 | 0.009714 | uM | 1.0291 | 3.916 | 100 | 101.45 |
| **1b** | cAMP | Antagonist | CNR1 | IC50 | >10 | uM | | | | 5.5843 |
| **1b** | cAMP | Antagonist | CNR2 | IC50 | >10 | uM | | | | 7.121 |
| **1d** | cAMP | Antagonist | CNR1 | IC50 | >10 | uM | | | | 2.1677 |
| **1d** | cAMP | Antagonist | CNR2 | IC50 | >10 | uM | | | | 0 |

**SD2. Control dose curves were performed for the GPCR Biosensor Assays to assess antagonistic action of GPR.** For antagonist assay, the cells were initially incubated with an agonist 20 mM or 25mM CP55940, for CB1 or CB2 respectively. After 30 min of incubation the cells were incubated with vehicle, a range of concentration for antagonist control (i.e. CB1 antagonist AM281 or CB2 antagonist SR144528), or a range of concentrations for 1D/1B fenchones. Data shown was normalized to the maximal and minimal response observed in the presence of antagonist control compound and vehicle respectively.

**SD3.** Cartilage Degradation Scoring Criteria.

| Score | | Observation |
|---|---|---|
| 0 | = | No degeneration. |
| 0.5 | = | Very minimal degeneration, within the zone less than 5% of the matrix has PG loss mainly with minor chondrocyte loss and little if any collagen matrix loss or damage. |
| 1 | = | Minimal degeneration, within the zone 5-10% of the matrix appears non-viable as a result of significant chondrocyte loss (greater than 50% of normal cell density). PG loss is usually present in these areas of cell loss and collagen matrix loss may be present. |
| 2 | = | Mild degeneration, within the zone 11-25% of the matrix appears non-viable as a result of significant chondrocyte loss (greater than 50% of normal cell density). PG loss is usually present in these areas of cell loss and collagen matrix loss may be present. |
| 3 | = | Moderate degeneration, within the zone 26-50% of the matrix appears non-viable as a result of significant chondrocyte loss (greater than 50% of normal cell density). PG loss is usually present in these areas of cell loss and collagen matrix loss may be present. |
| 4 | = | Marked degeneration, within the zone 51-75% of the matrix appears non-viable as a result of significant chondrocyte loss (greater than 50% of normal cell density). PG loss is usually present in these areas of cell loss and collagen matrix loss may be present. |
| 5 | = | Severe degeneration, within the zone 76-100% of the matrix appears non-viable as a result of significant chondrocyte loss (greater than 50% of normal cell density). PG loss is usually present in these areas of cell loss and collagen matrix loss may be present. |

**SD4.** Osteophyte Score.

| Score | | Observation | | |
|---|---|---|---|---|
| **0** | = | None | = | Less than 200 µm. |
| 1 | = | Small | = | 200-299 µm. |
| 2 | = | Medium | = | 300-399 µm. |
| 3 | = | Large | = | 400-499 µm. |
| 4 | = | Very Large | = | 500-599 µm. |
| 5 | = | Very Large | = | ≥600 µm. |

**SD5.** Medial Tibial Bone Damage Scoring Criteria.

| Score | | Observation |
|---|---|---|
| 0 | = | No changes. |
| 1 | = | Increased basophilia at tidemark, no fragmentation of tidemark, no marrow changes or if present minimal and focal, affects less than 10% of linear width of tidemark. |
| 2 | = | Increased basophilia at tidemark, minimal to mild focal fragmentation of calcified cartilage of tidemark affects approximately 1-10% of the linear width of the tidemark, mesenchymal change in marrow involves up to 1/4 of total area but generally is restricted to subchondral region under lesion, no collapse of cartilage into epiphyseal bone. |
| 3 | = | Increased basophilia at tidemark, mild to moderate focal or multifocal fragmentation or loss of calcified cartilage/subchondral bone (multifocal) affects 11-25% of linear width of tidemark, mesenchymal change in marrow is up to 3/4 of total area, areas of marrow chondrogenesis may be evident with focal collapse of articular cartilage into epiphyseal bone less than 200 μm deep. |
| 4 | = | Increased basophilia at tidemark, marked to severe fragmentation and or loss of calcified cartilage/subchondral bone affects 26-50% of linear width of tidemark, marrow mesenchymal change involves up to 3/4 of area and articular cartilage may have collapsed into the epiphysis to a depth of 200-350 μm or less from tidemark. |
| 5 | = | Increased basophilia at tidemark, marked to severe fragmentation and or loss of calcified cartilage/subchondral bone affects greater than 50% of the width of the tidemark, marrow mesenchymal change involves up to 3/4 of area and articular cartilage may have collapsed into the epiphysis to a depth of greater than 350 μm from tidemark. |

**SD6.** Synovitis scoring.

| Score | | Observation |
|---|---|---|
| 0 | = | Normal synovium. |
| 0.5 | = | Very minimal synovitis (generally focal or scattered minimal diffuse). |
| 1 | = | Minimal synovitis (generally focal or scattered minimal diffuse). |
| 2 | = | Mild synovitis (multifocal to confluent areas of mild mononuclear cell infiltration). |
| 3 | = | Moderate synovitis (confluent areas of moderate mononuclear cell infiltration). |
| 4 | = | Marked synovitis (confluent areas of marked mononuclear cell infiltration). |
| 5 | = | Severe synovitis (confluent areas of severe mononuclear cell infiltration). |

**SD7.** Raw Data (Excel Sheet) for MMT Experiment in Lewis Rat.
