# Supplementary material for: A Fenchone Derivative Effectively Abrogates Joint Damage Following Post-Traumatic Osteoarthritis in Lewis Rats
Source: Cells. 2022 Dec 16;11(24):4084. doi: 10.3390/cells11244084 (PMC9777073; doi:10.3390/cells11244084)
Supplement: Supplementary file 1 [file cells-11-04084-s001.zip › Suppl. Data_7.pdf]

SD7

Treatment group	Animal no.	Change in Body Weight Day -4 to 28	Day 4	Day 7	Day 14	Day 28	Day 4-28	Medial Tibial Cartilage Degeneration Score (0-5)				Medial Tibial Cartilage Degeneration Depth Ratio				Synovitis (0-5)	Osteophyte Measurement (µm)	Osteophyte Score (0-5)	Bone Damage (0-5)
			Weight Bearing Difference (Left-Right, grams)	Weight Bearing Difference (Left-Right, grams)	Weight Bearing Difference (Left-Right, grams)	Weight Bearing Difference (Left-Right, grams)	Area Under the Curve (AUC)	Zone 1 (outside)	Zone 2 (midside)	Zone 3 (inside)	Total of 3 zones	Zone 1 (outside)	Zone 2 (midside)	Zone 3 (inside)	Total of 3 zones				
Vehicle - Sham IA, 7x (D4, 7, 10, 14, 17, 21, 24)	1	46	-29.53	-9.94	17.96	1.59	105.72	0	0	0	0	0	0	0	0	0.5	0	0	0
	2	54	55.15	2.11	8.5	10.58	256.59	0	0	0	0	0	0	0	0	0.5	0	0	0
	3	50	23.5	-19.39	4.02	-32.65	-248.04	0	0	0	0	0	0	0	0	0.5	0	0	0
	4	58	7.04	-2.17	-14.89	0.51	-153.07	0	0	0.5	0.5	0	0	0.03	0.01	0.5	0	0	0
	5	57	21.65	14.91	-1.02	38.11	363.09	0	0	0	0	0	0	0	0	0.5	0	0	0
Vehicle - MMT IA, 7x (D4, 7, 10, 14, 17, 21, 24)	1	56	18.9	5.52	21.13	-4.55	245.97	3	2	0.5	5.5	0.59	0.17	0.07	0.27	1	400	3	2
	2	57	51.33	19.6	13.97	-19.53	184.97	5	2	0.5	7.5	1	0.15	0.07	0.41	1	600	5	3
	3	33	33.39	99.53	29.42	58.12	1263.49	1	1	0.5	2.5	0.12	0	0.03	0.05	0.5	500	4	0
	4	52	22.39	-3.75	31.95	32.21	575.78	4	1	0.5	5.5	1	0.05	0.06	0.37	0.5	340	2	2
	5	49	35.88	35.78	-13.6	13.37	183.51	2	1	0.5	3.5	0.47	0.05	0.03	0.18	1	450	3	1
	6	48	10.58	10.98	-7.02	2.16	12.18	1	0.5	0	1.5	0.17	0	0	0.06	0.5	440	3	2
	7	44	-4.55	33.44	-21.52	16.57	50.4	3	2	0.5	5.5	0.71	0.33	0.08	0.38	0.5	380	2	2
	8	38	84.47	93.56	40.12	33.64	1251.25	4	1	0.5	5.5	0.92	0.05	0.07	0.35	0.5	320	2	2
	9	38	19.26	55.18	-12	43.06	480.21	1	2	0	3	0.13	0.15	0	0.09	0.5	440	3	2
	10	53	48.56	20.12	49.87	58.82	1108.82	5	2	0.5	7.5	1	0.54	0	0.51	1	600	5	2
	11	75	53.07	-0.78	24.5	-3.41	309.09	4	2	0.5	6.5	0.93	0.09	0.03	0.35	0.5	600	5	4
	12	54	29.35	25.48	-12.76	-23.75	-128.81	4	2	0.5	6.5	0.67	0.35	0.03	0.35	0.5	500	4	3
	13	33	5.07	49.21	52.71	27.4	998.91	4	1	0.5	5.5	0.86	0.13	0.06	0.35	1	400	3	2
	14	35	51.91	37.16	-2.45	46.07	560.43	4	2	0	6	0.8	0.4	0	0.4	0.5	360	2	1
	15	57	35.43	38.11	53.95	-1.05	802.82	3	1	0.5	4.5	0.8	0.14	0.03	0.32	0.5	550	4	2
Fenchone 1D LOW (8 ug/knee) IA, 7x (D4, 7, 10, 14, 17, 21, 24)	1	42	76.56	4.6	38.56	-22.2	387.32	1	1	0	2	0.18	0.09	0	0.09	2	400	3	1
	2	65	45.32	34.9	69.72	50.94	1331.12	4	2	0.5	6.5	0.87	0.14	0.07	0.36	2	500	4	1
	3	82	33.06	47.85	22.33	30.5	736.81	4	3	0.5	7.5	1	0.75	0.07	0.61	2	800	5	4
	4	48	53.36	11.28	30.66	45.5	776.87	4	2	0	6	0.93	0.32	0	0.42	1	350	2	3
	5	33	33.8	29.06	29.61	6.68	553.67	4	1	0.5	5.5	0.31	0.23	0.06	0.2	1	400	3	2
	6	69	42.51	44.11	43.94	-28.3	547.59	5	3	0	8	0.8	0.59	0	0.46	2	800	5	4
	7	44	34.59	34.35	57.15	101.44	1533.79	4	2	0.5	6.5	0.87	0.23	0.04	0.38	2	360	2	1
	8	54	27.2	5.87	-8.53	34.43	221.6	4	3	0.5	7.5	0.73	0.72	0.08	0.51	1	550	4	2
	9	61	68.56	36.82	67.54	29.33	1201.42	1	0.5	0	1.5	0.47	0.11	0	0.19	1	500	4	1
	10	55	40.25	8.65	28.13	36.56	654.91	2	1	0	3	0.4	0.15	0	0.18	1	500	4	1
	11	59	-8.73	-10.19	26.45	11.47	293.97	3	2	0.5	5.5	0.17	0.15	0.04	0.12	1	300	2	2
	12	84	15.89	29.58	11.12	39.05	561.85	3	2	0	5	0.8	0.18	0	0.33	1	650	5	2
	13	56	-2.54	43.39	68.29	67.88	1405.35	2	1	0.5	3.5	0.05	0.15	0.07	0.09	0.5	350	2	1
	14	62	40.95	25.25	70.5	85.37	1525.52	4	2	0	6	0.87	0.09	0	0.32	1	500	4	3
	15	67	30.82	35.7	9.82	33.3	560.94	5	2	0	7	1	0.07	0	0.36	1	500	4	2
Fenchone 1D HIGH (24 ug/knee) IA, 7x (D4, 7, 10, 14, 17, 21, 24)	1	39	24.81	8.9	5.5	62.71	578.44	2	1	0	3	0.2	0.12	0	0.11	1	240	1	1
	2	53	26.88	5.29	1.74	5.19	121.37	1	1	0.5	2.5	0.13	0.12	0.04	0.1	1	360	2	1
	3	41	14.19	-12.02	26.36	97.51	920.54	1	0.5	0.5	2	0.47	0.07	0.04	0.19	1	300	2	1
	4	51	41.08	52.16	25.08	8.1	642.46	5	2	0	7	1	0.12	0	0.37	1	500	4	3
	5	40	43.75	40.75	26.94	76.76	1089.57	2	1	0	3	0.21	0.05	0	0.09	2	300	2	1
	6	56	72.41	15.63	8.55	-47.1	-53.16	3	2	0	5	0.22	0.15	0	0.12	1	500	4	2
	7	17	-15.54	22.84	17.71	-3.71	250.88	3	1	0	4	0.53	0.12	0	0.22	1	600	5	3
	8	51	24.51	40.38	-35.94	41.79	153.83	3	1	0.5	4.5	0.2	0.25	0.03	0.16	0.5	450	3	3
	9	55	35.99	51.36	14.67	28.36	663.34	1	1	0.5	2.5	0.1	0.2	0	0.1	1	550	4	1
	10	58	-1.86	26.28	17.71	51.9	677.87	4	1	0.5	5.5	0.87	0.14	0.07	0.36	1	450	3	2
	11	29	48.19	16.34	57.76	49.86	1109.49	1	1	0	2	0.13	0	0	0.04	0.5	400	3	1
	12	41	130.7	71.02	56.48	23.82	1310.93	1	0.5	0.5	2	0.4	0	0.03	0.14	0.5	220	1	2
	13	64	-34.5	27.62	7.36	-29.21	-40.84	5	1	0.5	6.5	1	0	0	0.33	0.5	450	3	3
	14	41	48.69	46.34	45.89	31.37	1006.17	1	1	0	2	0.17	0	0	0.06	0.5	200	1	1
	15	80	44.92	12.34	14.87	7.19	335.55	1	1	0	2	0.17	0.14	0	0.1	1	350	2	2
FGF-18 (3 ug/knee) IA, 3x (D7, 14, 21)	1	33	34.24	45.46	45.87	73.26	1273.12	2	1	0.5	3.5	0.41	0.45	0.04	0.3	4	800	5	2
	2	55	24.5	12.24	35.69	54.47	853.99	2	1	0.5	3.5	0.18	0.12	0.03	0.11	4	900	5	3
	3	50	38.73	28.37	39.83	151.99	1682.09	1	0.5	0.5	2	0.08	0.07	0.04	0.06	4	700	5	3
	4	46	54.3	-26.92	62.53	144.43	1614.43	2	0.5	0.5	3	0.87	0.06	0.04	0.32	4	800	5	3
	5	22	52.64	24.79	78.26	65.11	1480.41	1	0.5	0.5	2	0.2	0.06	0.04	0.1	4	400	3	2
	6	43	40.78	17.76	48.46	170.33	1851.11	1	1	1	3	0.07	0.12	0.06	0.09	4	600	5	2
	7	35	61.27	28.71	62.76	154.56	1976.36	2	1	0.5	3.5	0.28	0.14	0.07	0.16	4	700	5	2
	8	42	17.73	15.66	-11.62	182.47	1260.18	2	1	0.5	3.5	0.45	0.21	0.06	0.24	4	800	5	0
	9	43	81.63	4.18	46.23	28.37	827.35	1	1	0.5	2.5	0.2	0.23	0.07	0.17	4	1200	5	2
	10	42	36.85	46.86	95.43	187.86	2606.61	1	1	0.5	2.5	0.14	0.1	0.09	0.11	4	700	5	0
	11	39	8.29	21.9	86	147.72	2058.98	1	1	0.5	2.5	0.18	0.27	0.04	0.16	4	900	5	2
	12	57	26.75	30.21	52.57	219.35	2278.61	3	1	0.5	4.5	0.12	0.09	0.04	0.08	4	700	5	3
	13	46	23.32	44.3	-56.7	150.92	717.57	1	1	0.5	2.5	0.09	0.29	0.12	0.17	4	700	5	1
	14	64	17.95	38.54	73.56	123.64	1857.49	3	2	0.5	5.5	0.23	0.33	0.09	0.22	4	700	5	1
	15	44	27.81	23.98	84.89	136.65	2009.51	1	1	1	3	0.13	0.2	0.07	0.13	4	700	5	2
